# From high-quality assistance to emergency orthopaedics during COVID-19 pandemic: a northern Italy scenario for sports medicine

**DOI:** 10.1007/s11332-020-00721-8

**Published:** 2021-02-16

**Authors:** Marco Bigoni, Filippo Maria Anghilieri, Massimiliano Piatti, NIcolò Zanchi, Marco Turati

**Affiliations:** 1 Orthopedic Department, San Gerardo Hospital, University of Milano-Bicocca, Via Pergolesi 33, 20900 Monza, Italy; 2 Department of Medicine and Surgery, University of Milano-Bicocca, 20900 Monza, Italy; 3 Transalpine Center of Pediatric Sports Medicine and Surgery, University of Milano-Bicocca - Hospital Couple Enfant, Monza (Italy), Grenoble France; 4 Residency Program in Orthopaedics and Traumatology, University of Milan, Milan, Italy; 5 Department of Paediatric Orthopaedic Surgery, Hospital Couple Enfant, Grenoble Alpes University, Grenoble, France

Dear Editor,

Northern Italy is currently facing deep clinical burdens related to the COVID-19 outbreak. The Lombardy region, which counts a sixth of the Italian population (10.08 million inhabitants), was hit much earlier than other regions and it is still the most affected one (Table 1). The first Italian infectious outbreak was registered in February 2020 in Codogno, Lombardy [1], and from that moment, the Region counted 28.8% of cases and 45.9% of deaths of the country as of October 27th.

**Table 1:** COVID-19 surveillance data from the Civil Protection Department of the Italian Government (updated 27 October 2020)

	Inhabitants (millions)	*n* cases	*n* deaths	Case-fatality ratio	Mortality (per 100 000)
Lombardy	10.08	162,968	17,310	10.62%	171.73
Italy	60.37	564,778	37,700	6.67%	61.29

The rapidly expanding severe acute respiratory syndrome coronavirus 2 (SARS-CoV-2) has forced national and regional Governments to advise orthopaedic surgeons to postpone elective scheduled surgeries and outpatient visits. Thousands of orthopaedic operations that were not considered to cause a significant harm to the patient were thus delayed. The vast majority of healthcare structures and resources have been allocated to cope with the COVID-19 emergency.

In the field of orthopaedic and trauma surgery in Lombardy region, two hospitals were identified as referral hubs for minor trauma and non-deferrable elective surgeries. An orthopaedic expert meeting was organized in the referral centres to define the list of all the pathologies that could be considered as emergencies. This list included generic descriptions of the diseases (Table 2), not limited to a specific joint, to allow a broad interpretation of the particular diagnosis in question [2].

**Table 2:** English translation of the list from Lombardy Region ordinance of 15th March 2020

List of orthopaedic clinical situation to intend as non-deferrable elective surgeries in Lombardy
Septic arthritis
Malignancies
Peri-articular tumors and/or neoplastic lesions with risk of pathological fractures
Musculoskeletal pathologies determining neurological deficit
Evolving arthropaties (avascular necrosis)
Acute traumatic tendon injuries
Dislocations after joint replacement
Aseptic loosening of joint implants
Joint locking due to articular loose bodies

Available from: https://www.regione.lombardia.it/wps/wcm/connect/af6d2303-9212-433e-abcb-ac5a77b48fe5/Decreto+3353.pdf?MOD=AJPERES&CACHEID=ROOTWORKSPACE-af6d2303-9212-433e-abcb-ac5a77b48fe5-n3DPlAT

It is evident that sports medicine and traumatology are poorly represented in the abovementioned list. As a result, almost all the surgeries have been postponed, with the exception of diseases which, if not treated promptly, would have led patients to a bad outcome, for example, bucket-handle meniscal tears or biceps and achilles tendon ruptures. Better outcomes appear to be associated with early surgery in different interventions like meniscal repair and ACL avulsion fixation [3]. Nevertheless, the centralization of the patients in two hubs, with consequent transferring and testing for SARS-CoV-2 issues, has led to a lengthening of the waiting lists, making it difficult to do the surgery with the optimal timing.

Moreover, patients who underwent surgery during, or immediately before, the infectious outbreak were not able to access to outpatients checkups as normally scheduled nor to follow a high-quality rehabilitation protocol. In fact, many rehabilitation infrastructures have been temporary converted to the care of COVID-19 patients, while others, according to quarantine restrictions, were locked down. Furthermore, swimming pools have been closed again in Lombardy with a significant delay in planning post-operative physical therapy in water environment.

According to the concept of social distancing, hospitals and surgeons were asked to reduce the burden of outpatient visits, only admitting non-deferrable cases. Therefore, most sports medicine diseases were not considered suitable for first orthopaedic consultation. Those patients may have resorted to self-medication, pending a specialist examination.

Another matter for sports medicine during COVID-19 pandemic concerns with patients already diagnosed for their disease, for whom in many cases it is difficult to undergo conservative treatments. It is above all the case of anterior knee pain, a common presenting complaint that can be due to many underlying causes and whose management is primarily non-operative. Physiotherapy is reported to be extremely effective, but it has recently been poorly available for the abovementioned lockdown.

In October 2020, we are witnessing the so-called “second wave of COVID-19”, confirming that we will have to live with this pandemic for months.

In the nearest future, therefore, with the progressive resumption of elective surgeries, it will be appropriate to carefully select both patients and type of surgery to limit the risk of infections and complications. The selection of prioritized patients should take into account several parameters such as COVID-19 exposure, age, American Society of Anesthesiologists (ASA) physical status classification system, comorbidities and socio-professional situation [4]. In the absence of well-established scientific criteria, sports surgery, being minimally invasive, appears to be safer than other elective orthopaedic surgeries and may be considered one of the firsts for resuming.

Moreover, this scenario has shown the need to implement territorial care. We strongly believe that telemedicine, consisting in the practice of caring for patients remotely, and home-based physiotherapy will be solutions increasingly used to reduce hospital access, which results fundamental for Public Health. Tele-rehab and home-based care are vital either to get to a surgery in better condition, in terms of muscle tone and articular mobility, or to recover more quickly after the procedure.

Cooperation between orthopaedic surgeons and general practitioners is essential for this continuity of care inside and outside the hospital.
